# New insights from Gorongosa National Park and Niassa National Reserve of Mozambique increasing the genetic diversity of *Trypanosoma vivax* and *Trypanosoma vivax-*like in tsetse flies, wild ungulates and livestock from East Africa

**DOI:** 10.1186/s13071-017-2241-2

**Published:** 2017-07-17

**Authors:** Carla MF Rodrigues, Herakles A Garcia, Adriana C Rodrigues, André G Costa-Martins, Carlos L Pereira, Dagmar L Pereira, Zakaria Bengaly, Luis Neves, Erney P Camargo, Patrick B Hamilton, Marta MG Teixeira

**Affiliations:** 1 Departamento de Parasitologia, Instituto de Ciências Biomédicas, Universidade de São Paulo, São Paulo, SP Brazil; 2 Departamento de Patología Veterinaria, Facultad de Ciencias Veterinarias, Universidad Central de Venezuela, Maracay, Aragua Venezuela; 3 National Administration of Conservation Areas, Ministry of Tourism, Maputo, Mozambique; 4 Wildlife Conservation Society, Niassa National Reserve, Maputo, Mozambique; 5 Independent researcher, Maputo, Mozambique; 6 Centre International de Recherche-Développement sur l’Elevage en zone Subhumide (CIRDES), Bobo-Dioulasso, Burkina Faso; 7 Centro de Biotecnologia, Eduardo Mondlane University, Maputo, Mozambique; 8 Department of Veterinary Tropical Diseases, Faculty of Veterinary Science, University of Pretoria, Pretoria, South Africa; 9 Biosciences, College of Life and Environmental Sciences, University of Exeter, Exeter, UK

**Keywords:** African animal trypanosomiasis, Wildlife, Tsetse fly, Diagnosis, Genotyping, Phylogeny, Taxonomy, Evolution

## Abstract

**Background:**

*Trypanosoma* (*Duttonella*) *vivax* is a major pathogen of livestock in Africa and South America (SA), and genetic studies limited to small sampling suggest greater diversity in East Africa (EA) compared to both West Africa (WA) and SA.

**Methods:**

Multidimensional scaling and phylogenetic analyses of 112 sequences of the glycosomal glyceraldehyde phosphate dehydrogenase (gGAPDH) gene and 263 sequences of the internal transcribed spacer of rDNA (ITS rDNA) were performed to compare trypanosomes from tsetse flies from Gorongosa National Park and Niassa National Reserve of Mozambique (MZ), wild ungulates and livestock from EA, and livestock isolates from WA and SA.

**Results:**

Multidimensional scaling (MDS) supported Tvv (*T. vivax*) and TvL (*T. vivax*-like) evolutionary lineages: 1) Tvv comprises two main groups, TvvA/B (all SA and WA isolates plus some isolates from EA) and TvvC/D (exclusively from EA). The network revealed five ITS-genotypes within Tvv: Tvv1 (WA/EA isolates), Tvv2 (SA) and Tvv3–5 (EA). EA genotypes of Tvv ranged from highly related to largely different from WA/SA genotypes. 2) TvL comprises two gGAPDH-groups formed exclusively by EA sequences, TvLA (Tanzania/Kenya) and TvLB-D (MZ). This lineage contains more than 11 ITS-genotypes, seven forming the lineage TvL-Gorongosa that diverged from *T. vivax* Y486 enough to be identified as another species of the subgenus *Duttonella.* While gGAPDH sequences were fundamental for classification at the subgenus, major evolutionary lineages and species levels, ITS rDNA sequences permitted identification of known and novel genotypes.

**Conclusions:**

Our results corroborate a remarkable diversity of *Duttonella* trypanosomes in EA, especially in wildlife conservation areas, compared to the moderate diversity in WA. Surveys in wilderness areas in WA may reveal greater diversity. Biogeographical and phylogenetic data point to EA as the place of origin, diversification and spread of *Duttonella* trypanosomes across Africa, providing relevant insights towards the understanding of *T. vivax* evolutionary history.

**Electronic supplementary material:**

The online version of this article (doi:10.1186/s13071-017-2241-2) contains supplementary material, which is available to authorized users.

## Background

Animal African trypanosomiasis (AAT) caused by *Trypanosoma* (*Duttonella*) *vivax* is a major challenge to livestock production in sub-Saharan Africa [1]. This species is a highly prevalent livestock pathogen across the tsetse belt (cyclical transmission) [2–5], as well as in tsetse-free areas [6, 7]. Mechanical transmission by other biting flies allows the spread of *T. vivax* across Africa and South America (SA) [8, 9].

*Trypanosoma vivax* infections vary in clinical signs and disease severity, and differences in pathogenicity have been linked to breeds of livestock, parasite strains, and geographical locations. In East Africa (EA), there is a wide variation in pathogenicity and lethality. While wild ruminants remain asymptomatic, thus acting as reservoirs, livestock species generally develop significant degrees of anaemia and a range of pathological changes. Severe hemorrhagic syndromes in cattle have been reported in Kenya and Uganda [10, 11]. Furthermore, pathological lesions in various organs and nervous signs were reported in cattle that were experimentally infected with Ethiopian isolates of unknown genotypes [6].

In West Africa (WA), *T. vivax* causes chronic and debilitating diseases in livestock [1, 12]. In endemic areas of SA, *T. vivax* infections are generally asymptomatic, with beef cattle and water buffalo usually developing chronic disease with very low parasitemia [8, 13]. However, we recently reported an outbreak in water buffaloes from the Venezuelan Llanos with high mortality, which is likely to have been induced by stressful conditions during a prolonged drought [13]. In the last decade, many outbreaks of acute disease have been reported in non-endemic SA regions, affecting naïve dairy cattle, sheep and horses, which exhibit signs of acute disease with high parasitemia, progressive haematological and neurological disorders, and often leading to death when left untreated [14–16].

Data from earlier studies revealed genetic similarities between SA (Colombian) and WA *T. vivax* [17, 18]. This finding was corroborated by phylogenetic studies suggesting that *T. vivax* was introduced into the New World *via* cattle imported from WA by European colonisers [8, 19, 20]. In contrast with the genetic homogeneity of isolates from SA [8, 13, 19, 21] and WA [4, 8, 22, 23], studies have unveiled greater genetic diversity among isolates from Tanzania, Mozambique (MZ), Kenya and Ethiopia [2, 7, 19, 24–27]. Sequences of gGAPDH from Tanzanian isolates obtained from tsetse flies diverged largely from those detected in cattle and nyala antelope from MZ, which were closer to WA/SA than to another EA *T. vivax* [25, 26]. In addition, ITS rDNA polymorphisms corroborated large polymorphism among isolates from Tanzania and MZ [2, 19, 24]. ITS sequences from Ethiopian *T. vivax* isolates from cattle clustered either with sequences of isolates from WA or other EA countries regardless of whether they were collected in areas infested by tsetse or not [7]. Despite the small genetic diversity, isolates of Nigerian cattle clustered with WA/SA genotypes [4]. Cathepsin L-like (CATL) sequences from *T. vivax* isolates from Zambia (South-Central Africa) clustered with either WA or divergent EA sequences while all isolates from Ghana clustered with those from WA [20, 21, 23].

Characterization of a limited sampling suggests a higher diversity of *T. vivax* in EA, and probable also in South-Central Africa than in WA/SA [2, 7, 19, 24–26]. However, the use of different markers and difficulty in obtaining DNA sequences from tsetse flies and wild animals that are suitable for phylogenetic analyses have hampered the evaluation of the extent of *T. vivax* diversity. To evaluate genetic repertoires, and any possible links between genotypes and geography, ungulate and vector species, outbreak and endemic areas, and clinical and pathological features, more comprehensive studies with larger sampling are still required. Data on trypanosome diversity in conservation areas are fundamental to wildlife conservation, to track the spread of parasites to bordering farming zones, and to monitor potential pathogenicity in livestock of trypanosome species/genotypes sustained by natural transmission cycles.

The present study aimed to assess the genetic repertoire of *T. vivax* in MZ and to infer phylogenetic relationships among EA, WA and SA *T. vivax* populations. To achieve this, we determined gGAPDH, and ITS rDNA sequences from a comprehensive sampling of *T. vivax* isolates from tsetse flies captured at the Gorongosa National Park (GNP) and Niassa National Reserve (NNR), and from wild ungulates and livestock from MZ. The sequences determined in the present study were then compared to those available from other EA countries, and with sequences obtained herein or in previous studies of *T. vivax* from livestock across WA and SA.

## Methods

### Wildlife reserves of Mozambique included in this study

**Fig. 1:**
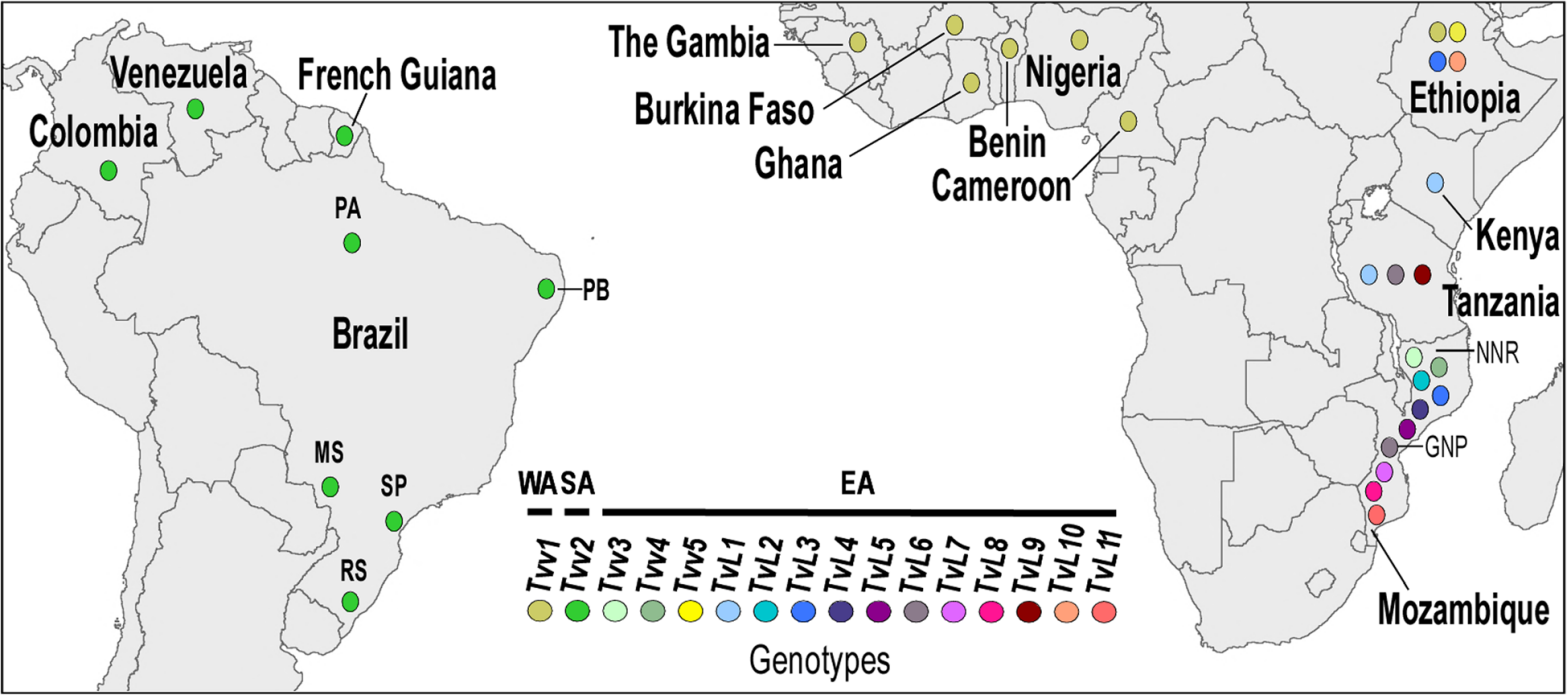
Geographical origins of *Trypanosoma vivax* isolates characterised in this study. Isolates from Mozambique were from tsetse flies captured at Gorongosa National Park (GNP) and Niassa National Reserve (NNR), and from blood samples of Cape buffalo, antelopes and livestock from other areas. Isolates from Tanzania and Ethiopia were from tsetse flies and cattle, respectively (sequences retrieved from GenBank). Cattle isolates from West Africa (WA) were from tsetse-infested regions, and livestock samples from South America (SA) were from endemic zones and outbreaks in non-endemic areas. Brazilian States: PA, Pará; PB, Paraíba; MS, Mato Grosso do Sul; SP, São Paulo; RS, Rio Grande do Sul

Studies on tsetse flies in MZ were conducted at the Gorongosa National Park (GNP) (18°45′58″S 34°30′00″E) and Niassa National Reserve (NNR) (12°08′35″S, 37°40′08″E). GNP comprises an area of ~4000 km^2^ (10,090 km^2^ including the buffer zone) located in the centre of MZ in the Province of Sofala, at the southern end of the Great African Rift Valley (Fig. 1). Regular seasonal flooding contributes to the variety of ecosystems in GNP, with grassland plains, savannah, floodplains and rain forests, surrounded by mountains and limestone cliffs. In the past, this area supported uniquely large numbers of large mammals, but they were drastically reduced by the long lasting civil war at the end of the twentieth century. A wildlife restocking program has been introduced, and large herbivores such as Cape buffalo, antelopes and elephants have been relocated from other Mozambican and South African parks. At the time of tsetse capture, GNP harboured large herds of antelopes (waterbucks, impalas, bushbucks, reedbucks, nyalas, kudus), Cape buffalo, an increasing number of elephants and hippopotamus, and a notable abundance of warthogs. Livestock is virtually absent within GNP, but goats are present in bordering areas.

NNR is a large wilderness area covering over 42,000 km^2^ located in the northern Provinces of Cabo Delgado and Niassa bordering Tanzanian Game Reserves (Fig. 1), allowing animal migration across the Trans-Frontier Conservation Area. NNR is mostly covered by miombo forest, open savannah and wetlands, with high wildlife diversity. There is a great concentration of elephants, antelopes (impala, kudu, sable, waterbuck, hartebeest, nyala, wildebeest, duiker and eland among others), Cape buffalo, hippopotamus, zebras, wild suids and great felines. GNP and NNR are home to a large diversity of small mammals, birds, amphibians and reptiles, including large populations of crocodiles. Game hunting, agricultural and livestock (mainly goats) occur in this area. NNR and GNP are separated by ~900 km.

### Tsetse fly collection, identification and microscopic screening for trypanosomes

Tsetse flies were captured in highly infested areas of GNP (2007, 2009 and 2012) and NNR (2013 and 2014) (Additional file 1: Table S1 and Additional file 2: Table S2) in the morning and in the afternoon using slow-moving vehicles to attract the flies that were then manually collected. Flies were cleaned twice by immersion in sterilised water, dried on filter paper, dissected, and guts microscopically examined for the presence of trypanosomes. Samples of guts and mouthparts of flies selected by microscopy, and from flies that had not been previously microscopically surveyed, all preserved in ethanol, were examined in this study. Tsetse flies preserved in ethanol (99.5%) were identified by morphology and cytochrome *c* oxidase subunit 1 (*cox*1) barcoding [28].

### Blood collection from wild and domestic ruminants in Africa and South America

Blood was collected from Cape buffalo in the GNP, from wildebeests and Cape buffalo from NNR, and from antelopes in a game reserve at the province of Sofala. Blood samples from livestock were from small herds of cattle and goats, collected in the provinces of Tete, Sofala (Centre) and Maputo (South) of MZ. Livestock was raised having rare contact with wild ruminants, except in areas bordering conservation areas and game reserves, where wild animals and livestock frequently interact (Fig. 1, Additional file 1: Table S1).

From 2008 to 2010, cattle blood samples from WA were collected in tsetse-infested areas in The Gambia, Burkina Faso, Ghana, Benin and Nigeria. In SA, *T. vivax* samples were obtained from cattle, water buffalo, sheep and horses from Venezuela, Brazil, Colombia and French Guiana (Fig. 1, Table 1, Additional file 2: Table S2). Venezuelan Llanos and Brazilian Amazonia and Pantanal are wetlands endemic for *T. vivax* with extensive cattle and buffalo production, where hematophagous flies are abundant throughout the year. Samples from cattle and sheep outbreaks were obtained from Northeastern Brazil, which is home to the largest herds of goats, sheep and donkeys in SA, and from outbreaks in Southeast and South Brazil [15, 16, 21].

**Table 1:** *Trypanosoma* isolates, host and geographical origin, and gGAPDH genetic groups and ITS rDNA genotypes

Isolate	Host species	Geographical origin		Year	gGAPDH group	ITS rDNA genotype
*Trypanosoma vivax*						
Y486	cattle	WA	Nigeria	1976	TvvA	Tvv1
TviBfMatorkou	cattle	WA	Burkina Faso	2008	TvvA	Tvv1
Gambia	cattle	WA	The Gambia	2009	TvvA	Tvv1
FN400714	cattle	WA	Gambia	–	TvvA	–
TviBan1	cattle	WA	Benin	2008	TvvA	Tvv1
FP9^a^	cattle	WA	Cameroon	–	TvvA	–
Dere091, KC92J28, KCA19J56	cattle	WA	Ghana	2008	TvvA	Tvv1
Desowitz^a^	cattle	WA	Nigeria	2005	TvvB	–
TviKang92	cattle	WA	Ghana	2008	TvvB	Tvv1
IL700^a^	cattle	WA	Nigeria	–	–	Tvv1
TviBfMene	cattle	WA	Burkina Faso	2008	–	Tvv1
TviBf:L445, Folonzo	cattle	WA	Burkina Faso	2008	–	Tvv1
TviBan1.2, Kommon, Bolonsi63	cattle	WA	Benin	2008	–	Tvv1
AF047500^a^	cattle	WA	–	–	TvvB	–
TviBrCa	cattle	SA	Brazil	2002	TvvA	–
TviBrSP2	cattle	SA	Brazil	2008	TvvB	Tvv2
TviBrMi	cattle	SA	Brazil	1997	TvvA	Tvv2
TviBr:Bov1, Po, RS2	cattle	SA	Brazil	1996/2000/ 2009	–	Tvv2
TviBrPA	cattle	SA	Brazil	2008/2007	–	–
TviVz: Ap, Anz	cattle	SA	Venezuela	2006	–	Tvv2
AF053744	cattle	SA	Venezuela	–	TvvA	Tvv2
TviColômbia	cattle	SA	Colombia	2014	TvvA	–
TviGuyane	cattle	SA	French Guiana	1986	TvvB	Tvv2
TviBr: PA, PB	water buffalo	SA	Brazil	2008/2009	TvvB	Tvv2
TviVzAp	water buffalo	SA	Venezuela	2015	TvvA	Tvv2
TviBr: PB, RP	sheep	SA	Brazil	2008/2009	TvvB	–
TviVzAp	sheep	SA	Venezuela	2006	–	Tvv2
TviBrRS1	horse	SA	Brazil	2009	TvvB	Tvv2
TviMzCb11	cattle	EA	Mozambique	2007	TvvB	–
TviMzCb12	cattle	EA	Mozambique	2007	TvvD	Tvv3
TviMzSoAbu21	cape buffalo	EA	Mozambique	2011	TvvB	–
TviMzNy	nyala	EA	Mozambique	2006	TvvD	Tvv4
TviMzG2115, 1686	tsetse fly	EA	Mozambique	2009	TvvD	Tvv4
TviMzG1926, 2172, 2175, 2194, 68, 215, 403, 405, 449, 464, 545	tsetse fly	EA	Mozambique	2012/2013	TvvB,C,D	–
TviMzG375	tsetse fly	EA	Mozambique	2007	TvvC	Tvv3
TviMzMa61	cattle	EA	Mozambique	2007	–	Tvv4
Fc-ET^b^	cattle	EA	Ethiopia	2012	–	Tvv5
4338 – ET^b^	cattle	EA	Ethiopia	2012	–	Tvv5
*T. vivax-like*: TvL Gorongosa						
IL3905	cattle	EA	Kenya	1986	TvLA	TvL1-G
TS06009^c^	cape buffalo	EA	Tanzania	2006	–	TvL1-G
FM164786-89^a^	tsetse	EA	Tanzania	2006/2007	TvLA	–
MZG87	tsetse fly	EA	Mozambique	2007	TvLA	TvL2-G
TviMzGnu12	wildebeest	EA	Mozambique	2013	TvLB	TvL3-G
4337-Et^b^	cattle	EA	Ethiopia	2012	–	TvL3-G, TvL10
TviMzCb3	cattle	EA	Mozambique	2007	TvLB	–
TviMzSoAbu21	cape buffalo	EA	Mozambique	2011	TvLB,C	–
TviMzG46	tsetse fly	EA	Mozambique	2012	TvLB	–
TviMzG1488	tsetse fly	EA	Mozambique	2009	TvLC	TvL4,6-G
TviMzG1477, 1375	tsetse fly	EA	Mozambique	2009	TvLC	TvL5-G
TviMzG1375, 2115	tsetse fly	EA	Mozambique	2009	TvLC,D	TvL5-G
TviMzG64,1585, 60, 403, 404, 433, 510	tsetse fly	EA	Mozambique	2009/2014	TvLC,D	TvL6-G
TviMzG719	tsetse fly	EA	Mozambique	2009	TvLC	–
TviMzG10, 1046	tsetse fly	EA	Mozambique	2012	TvLD	–
TviMzG25	tsetse fly	EA	Mozambique	2012	–	TvL6-G
TS07154–TZ^c^	waterbuck	EA	Tanzania	2007	–	TvL6-G
TviMzG474, 571, 1642, 1901	tsetse fly	EA	Mozambique	2007/2009	TvLC, D	TvL7-G
TviMzG417, 634, 24	tsetse fly	EA	Mozambique	2007/2009/2012	TvLD	TvL7-G
TviMzG346, 406, 1999, 62	tsetse fly	EA	Mozambique	2007/2009/2012	–	TvL6,7-G
TviMzG375	tsetse fly	EA	Mozambique	2007	–	TvL7-G, TvL8
*T. vivax-like*						
TS07214-TZ^c^	giraffe	EA	Tanzania	2007	–	TvL10
TviMzG571, 1686, 10, 68	tsetse fly	EA	Mozambique	2007/2012	TvL	–
FM164790^a^	tsetse fly	EA	Tanzania	2006/2007	TvL	–

*Abbreviations*: *WA* West Africa, *SA* South America, *EA* East Africa. Lineages: Tvv, *T. vivax*; TvL, *T. vivax*-like; TvL-G, TvL-Gorongosa – not determined

^a^Sequences from [25]

^b^Sequences from [7]

^c^Sequences from [2]

### Molecular identification of trypanosomes in ungulate blood samples and tsetse flies

Aliquots (~1.0 ml) of blood were collected using EDTA-treated tubes, preserved in ethanol and employed for DNA preparation as previously described [8, 20, 21]. *Trypanosoma vivax* diagnosis in ungulates was conducted using a *T. vivax*-specific PCR (TviCATL-PCR) [20]. Tsetse flies (guts and mouthparts) were tested using the fluorescent fragment length barcoding (FFLB) and TviCATL-PCR methods [29, 30]. We previously standardised this method using reference species to define the fluorescence peak profiles for the agents of African Animal Trypanosomiasis using an ABI3500 Genetic Analyser (Garcia et al. in preparation). Tsetse DNA samples that were positive for *T. vivax* using FFLB were submitted to whole genome amplification (WGA) using the REPLI-g UltraFast Mini Kit (Qiagen, Hilden, Germany).

### PCR amplification and sequencing of gGAPDH and ITS rDNA sequences

PCR amplification of gGAPDH sequences from DNA from tsetse flies and blood samples was conducted using a nested PCR method [31]. Each amplified DNA fragment was cloned, and a different number of clones was sequenced for each sample. This procedure was necessary, as infections with multiple trypanosome species and genotypes are common in tsetse flies. Sequences of ITS rDNA (ITS1 + 5.8S + ITS2) were amplified by PCR (~600 bp), cloned and sequenced (~5–7 clones from each sample) using primers and PCR conditions described previously [19]. PCR-amplification of ITS rDNA sequences from *T. vivax* and *T. vivax*-like obtained from tsetse flies had a lower sensitivity compared with FFLB and required cloning and sequencing of several clones from each sample. Sequences obtained were deposited in GenBank (Table 1, Additional file 1: Table S1, Additional file 2: Table S2).

### Comparison of gGAPDH and ITS rDNA sequences by multidimensional scaling (MDS) analysis

To provide a visual representation of the level of similarity across sequences in the dataset, we carried out multidimensional scaling (MDS) analyses plotted in 2D and 3D using the R software platform (R Development Core Team, http://www.R-project.org). MDS was performed using the *Bios2mds* package [32].

### Phylogenetic analyses of gGAPDH and ITS rDNA sequences

The whole gGAPDH sequences dataset was aligned and identical sequences from the same sample were removed from the final alignment, which also included sequences from GenBank of *T. vivax* and species of the subgenera *Trypanozoon (T. brucei brucei*, *T. b. rhodesiense*, *T. B. gambiense* and *T. evansi*), *Pycnomonas* (*T. suis*) and *Nannomonas* (*T. congolense* of the Savannah, Forest and Kilifi groups*, T. simiae*, *T. simiae* Tsavo and *T. godfreyi*). The final alignment was analysed using maximum parsimony (P) and the program PAUP*4.0b10, and maximum likelihood (ML) using RAxML with GTRGAMMA (500 maximum parsimony starting trees), model parameter estimated in RAxML over the duration of the tree search, and nodal support estimated with 500 bootstrap replicates.

Sequences of ITS rDNA (ITS1 + 5.8S + ITS2) obtained from DNA of tsetse and blood samples were aligned with sequences available in GenBank (Additional file 1: Table S1; Additional file 2: Table S2). The alignment was manually adjusted, and network split decomposition was inferred using the Neighbor-Net method with Kimura 2 parameters implemented as previously described [33, 34]. Internode support was estimated with 100 bootstrap replicates, using the parameters optimised for network inferences.

## Results

### Tsetse fly identification and prevalence of *T. vivax* and *T. vivax*-like determined by fluorescent fragment length barcoding (FFLB)

Barcoded tsetse flies from GNP and NNR were identified as *Glossina morsitans morsitans,* which was the predominant species in both studied areas accounting for 85.5% of all flies captured, plus lower frequency (14.5%) of *Glossina pallidipes*. Similar prevalence of *T. vivax* and *T. vivax*-like was found for the two species of tsetse flies (Garcia et al. in preparation).

Prevalence rates of *T. vivax* and *T. vivax*-like in 151 fly mouthparts examined by FFLB ranged from 19.2% (29 positive flies of 151 examined) in GNP to 28.6% (93 positive flies of 325 examined) in NNR, and was not significantly different between the two-tsetse species (Garcia et al. in preparation). Altogether, results from GNP and NNR surveys unveiled FFLB profiles that were compatible with *T. vivax* in the mouthparts of 122 flies (25.6%) out of 476 flies examined, including 12 (2.5%) flies positive for *T. vivax* and *T. vivax*-like in both mouthparts and guts, and 8 (1.6%) flies showing only positive guts*.* Detection of *T. vivax* in fly guts suggests that parasites can be detected using sensitive diagnostic methods from a fresh blood meal or from contamination during fly dissection. However, as expected, FFLB of most gut samples did not reveal *T. vivax* or *T. vivax*-like (Garcia et al. in preparation). Isolates considered positive for *T. vivax* differed in some peaks of the whole FFLB profiles. However, *Duttonella* exclusive fluorescent peaks were always present.

### Diagnosis of *T. vivax* and *T. vivax*-like in tsetse flies and ungulates by TviCATL-PCR

Analysis of MZ tsetse flies using TviCATL-PCR revealed infections in 43 out of 235 tsetse flies (18.2%) tested using this method, demonstrating that the sensitivity of this PCR method is inferior, but rather comparable to FFLB (25.6%, 122 out of 476 flies tested). In addition, TviCATL-PCR detected infection in 10 Cape buffalo (out of 98 animals tested) and one wildebeest (out of 15 animals). In Sunni antelopes (*n* = 5), reedbuck (*n* = 2) and warthogs (*n* = 7) the results were negative.

### Comparison of *Duttonella* trypanosomes from East, West and South America by MDS and phylogenetic analyses of gGAPDH sequences

**Fig. 2:**
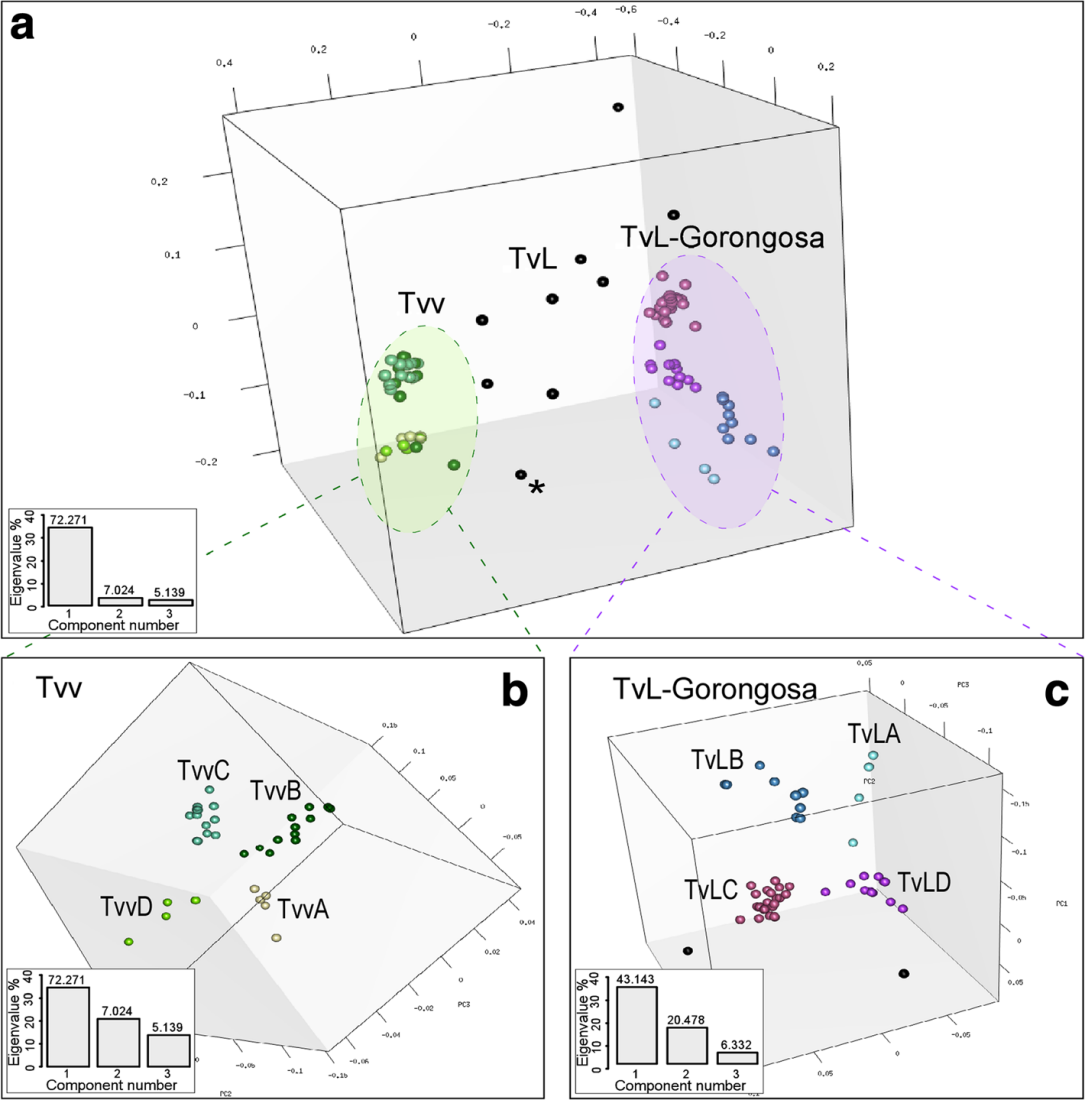
Multidimensional scaling (MDS) analysis of gGAPDH sequences from *Trypanosoma vivax* and *T. vivax*-like. **a** Space distribution of gGAPDH sequences showing two main clusters, Tvv and TvL-Gorongosa, defined by three components (PC1-PC3) and K-means method using the pairwise alignment of 112 gGAPDH sequences. **b** MDS of Tvv dataset evidencing the highly cohesive cluster TvvA/B of isolates from West Africa (WA) and South America (SA) plus a few from EA, and the more divergent TvvC-TvvD arrangement of sequences from East Africa (EA). **c** MDS of TvL dataset showing TvLA-D and highly divergent sequences (black circles) that remained un-clustered. Eigenvalue for all components is graphically represented for each analysis. GenBank accession numbers of gGAPDH sequences and trypanosome details are presented in Additional files 1 and 2

In previous studies, only five gGAPDH sequences of *T. vivax* were compared. These were distributed into the former groups: A and B, comprising Tanzania isolates; and group C, formed by isolates from SA (Brazil), WA (Nigeria) and EA (MZ) [25, 26]. In the present study, 65 out of 101 high-quality gGAPDH sequences (50 from GNP and 15 from NNR), obtained from 33 tsetse flies selected by FFLB and representative of the unveiled polymorphism, were compared by MDS. This analysis revealed a highly cohesive cluster, henceforward referred to as Tvv (the lineage including the reference *T. vivax* Y486), and a broader assemblage of sequences referred to as Tv-like (TvL) lineage, comprising a consistent cluster forward referred as TvL-Gorongosa, formed by groups A-D of isolates diverging from *T. vivax* Y486 by 4.8 to 5.7% (average of 5.2%) of gGAPDH sequence divergence. In addition, ~8.0% of sequences obtained from GNP and NNR tsetse flies remained unclustered, suggesting that they represent additional TvL lineages (Fig. 2a).

To obtain better resolved MDS clustering for the assessment of inter- and intra-cluster diversity, the dataset from each main cluster was analysed separately (Fig. 2b, c). Tvv unveiled four groups distributed in two major assemblages; one composed of TvvA clustered with TvvB, and the other composed of TvvC tightly related to TvvD (Fig. 2b). Average divergences of gGAPDH sequences were small: 0.2% for TvvA, 0.7% for TvvB, 0.5% for TvvC, and 0.8% for TvvD. TvvA (isolates from SA and WA livestock) diverged 0.7% from TvvB (WA and SA cattle isolates, and MZ cattle, buffalo and tsetse), 1.3% from TvvC (MZ tsetse), and 0.7% from TvvD (cattle, tsetse and nyala from MZ).

**Fig. 3:**
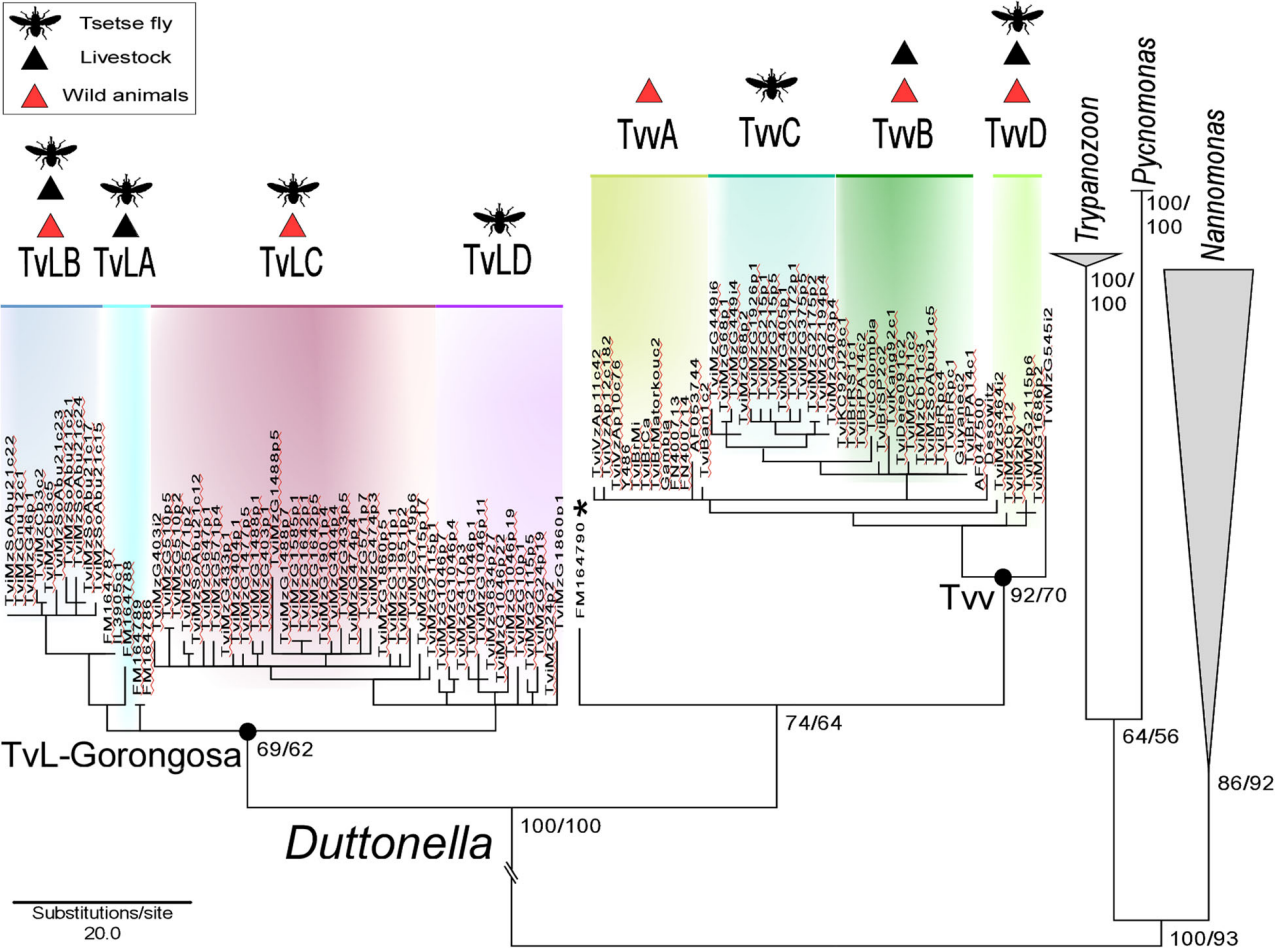
Phylogenetic analysis of *Trypanosoma vivax* and *T. vivax*-like based on gGAPDH sequences. Phylogenetic (parsimony) tree (609 characters) strongly supporting all trypanosomes of the subgenera *Duttonella* forming a monophyletic assemblage basal to the clade of pathogenic trypanosome comprising the species of the subgenera *Trypanozoon*, *Pycnomonas* and *Nannomonas.* Tvv and TvL-Gorongosa lineages were strongly supported and each one composed of four main clusters, TvvA-D and TvLA-D, respectively. One sequence from Tanzania (*) was placed between the two lineages, suggesting additional TvL lineages in EA

The highly divergent TvL sequences were arranged in four main clusters: TvLA (formerly referred to as “group A” [25] that clustered with the newly-identified group TvLB in addition to the cluster composed of TvLC and TvLD; altogether supporting TvL-Gorongosa lineage (Figs. 2 and 3). TvLA was closely related (1.5% of gGAPDH divergence) to TvLB, while TvLC tightly clustered with TvLD (2.0% of divergence). TvLA encompasses isolates from Tanzania and Kenya, and TvLB-D isolates are exclusively from MZ. TvLC and TvLD represented the predominant genotypes in tsetse flies from GNP and NNR (Figs. 2 and 3, Table 1).

The two major clusters were not arranged by order of wild or domestic hosts, tsetse species or date of collection. However, TvL-Gorongosa exclusively included isolates from EA, and Tvv comprised all samples from WA and SA, as well as some sequences from MZ and Ethiopia (Fig. 1, Table 1). Despite consistent results and high correspondence between MDS clusters and evolutionary lineages uncovered by gGAPDH sequences, with moderate support, the relationships among trypanosomes within the two lineages were not well-resolved, neither in P (Fig. 3) nor ML (data not shown) phylogenetic inferences using gGAPDH sequences. However, taken together, MDS and phylogenetic analyses allowed for the general delineation of 8 gGAPDH genetic groups as summarised in Table 1 (detailed information of each sample, host species and geographical origin are presented in Additional file 1: Table S1 and Additional file 2: Table S2).

In addition to the sequences that clustered into TvL-Gorongosa, 8 sequences from MZ tsetse flies remained isolated, although they were consistently included into the broad TvL assemblage. Similarly, a single sequence from Tanzanian tsetse, which was assigned to “group B” [25], largely diverged from all other sequences, however, its closest relatives appear to be members of Tvv.

Our analyses corroborated gGAPDH sequences as valuable markers for the identification of species, lineages and major intraspecific groups, but were unable to distinguish between the very closely related *T. vivax* genotypes circulating in SA, WA and some EA countries.

### Genotyping and relatedness of *Trypanosoma vivax* and *Trypanosoma vivax*-like ITS rDNA sequences assessed by MDS

To further assess intra-lineage genetic diversity, we compared 263 ITS rDNA sequences determined herein with all available GenBank sequences (17) from MZ, Kenya, Tanzania and Ethiopia. The analysis included a large set of ITS sequences from WA (59 sequences from cattle) and SA (91 sequences from cattle, sheep, water buffalo and horses), most of them determined in the present study (Table 1, Additional file 1: Table S1, Additional file 2: Table S2). Analysis of ITS sequences that were representative of the whole genetic diversity, as well as of the geographical and host-species ranges revealed an extensive polymorphism in EA compared to WA/SA. Although the alignment of ITS sequences from EA isolates showed many ambiguities due to the extensive polymorphisms, blocks of ITS1 and ITS2 rDNA nucleotides characteristic for each group/genotype were detected (Additional file 3: Figure S1).

**Fig. 4:**
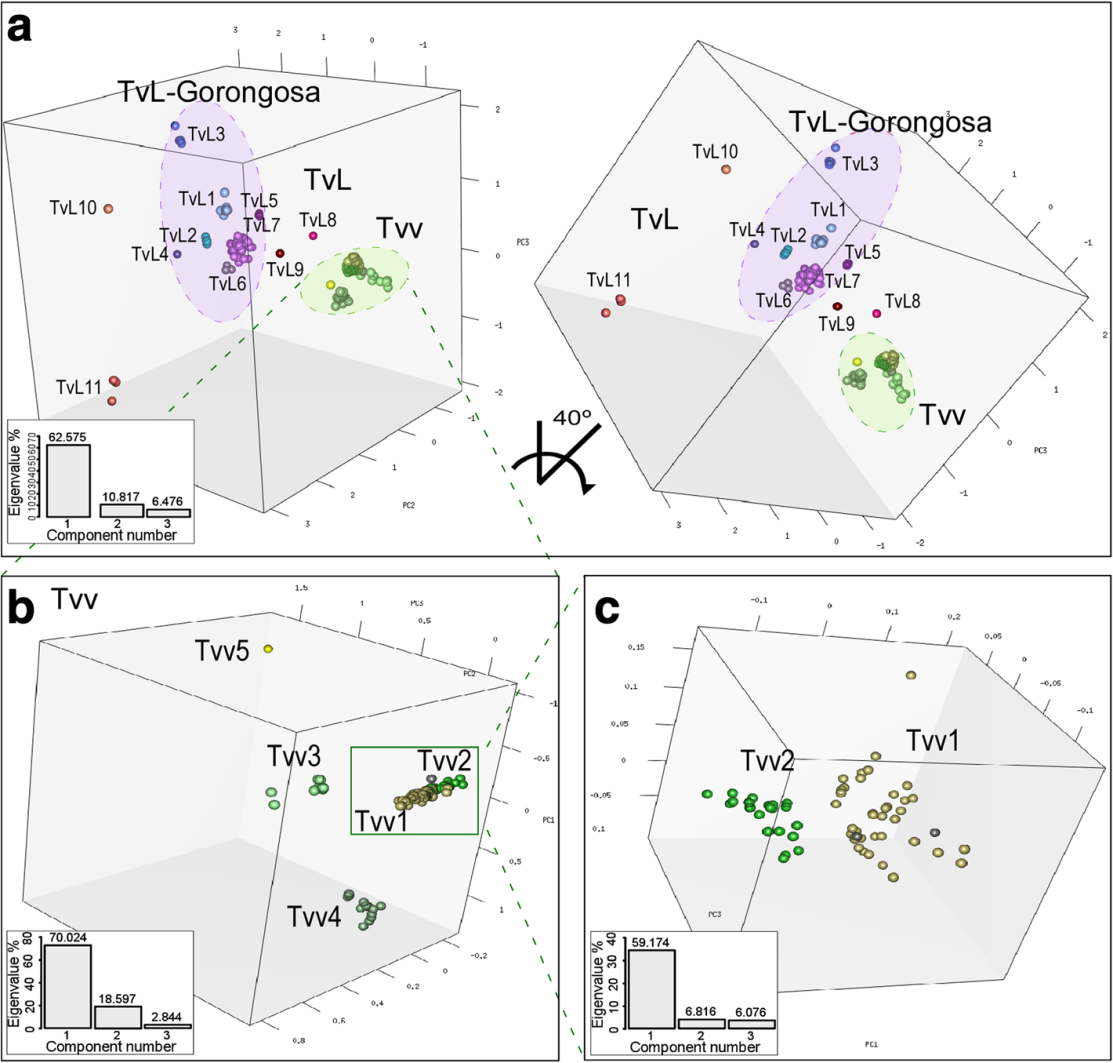
Multidimensional scaling (MDS) plot of ITS rDNA from *Trypanosoma vivax* and *T. vivax*-like. **a** Space distribution of 263 ITS sequences from tsetse flies and blood samples inferred with three components (PC1, PC2 and PC3) and K-means MDS method. The analysis support Tvv and TvL-Gorongosa as the major clusters, in addition to unclustered sequences. **b** MDS restricted to Tvv formed by sequences from South America (SA), West Africa (WA), Mozambique and Ethiopia (EA). **c** MDS restricted to SA/WA dataset unveiling genotypes Tvv1 from WA (plus two sequences from Ethiopia in grey circles), Tvv2 (SA), Tvv3-Tvv4 from MZ, and the Ethiopian Tvv5. MDS of TvL sequences showing a broad array of genotypes TvL1-TvL7 forming the lineage TvL-Gorongosa, in addition to highly divergent unclustered sequences. Eigenvalues are graphically represented for each analysis. Accession numbers in GenBank of ITS sequences are showed in Additional files 1 and 2

To investigate relatedness among the isolates, ITS sequences were submitted to MDS (Fig. 4). The results support a highly cohesive cluster of sequences representing Tvv, a broader arrangement of sequences supporting TvL-Gorongosa, and unclustered sequences representing other TvL lineages (Fig. 4a). In contrast to quite conserved gGAPDH sequences, large polymorphisms of ITS rDNA supported the separation between Tvv1 and Tvv2 genotypes. Tvv1clustered all WA isolates, while Tvv2 was restricted to SA, as unveiled by 3D MDS restricted to the whole set of SA/WA sequences (Fig. 4b) or comprising exclusively Tvv1 and Tvv2 (Fig. 4c) datasets. More relevant polymorphisms were observed within Tvv1 compared to Tvv2. Nevertheless, Tvv1 and Tvv2, which are to date restricted to livestock, share a greater degree of similarity between themselves compared with Tvv3 and Tvv4 of EA isolates from livestock, tsetse and wild buffalo. Two sequences from Ethiopian cattle clustered with Tvv1 and a single sequence was assigned to the new Tvv5 genotype (Fig. 4b). Tvv1 and Tvv2 are both genotypes of long-range dispersal linked to livestock: Tvv1 is the most geographically dispersed genotype occurring from the Gambia (WA) to Ethiopia (EA); Tvv2 is widespread in SA and was identified in Brazil, Venezuela, Colombia and French Guiana (Fig. 1).

Clustering of TvL ITS sequences analysed by 3D MDS support the highly heterogeneous lineage TvL-Gorongosa as defined by gGAPDH sequences (Fig. 4c, Table 1). Furthermore, ~8.0% of EA sequences from tsetse flies did not consistently nest within any cluster (Fig. 4a).

### ITS rDNA network of *T. vivax* and *T. vivax*-like isolates

**Fig. 5:**
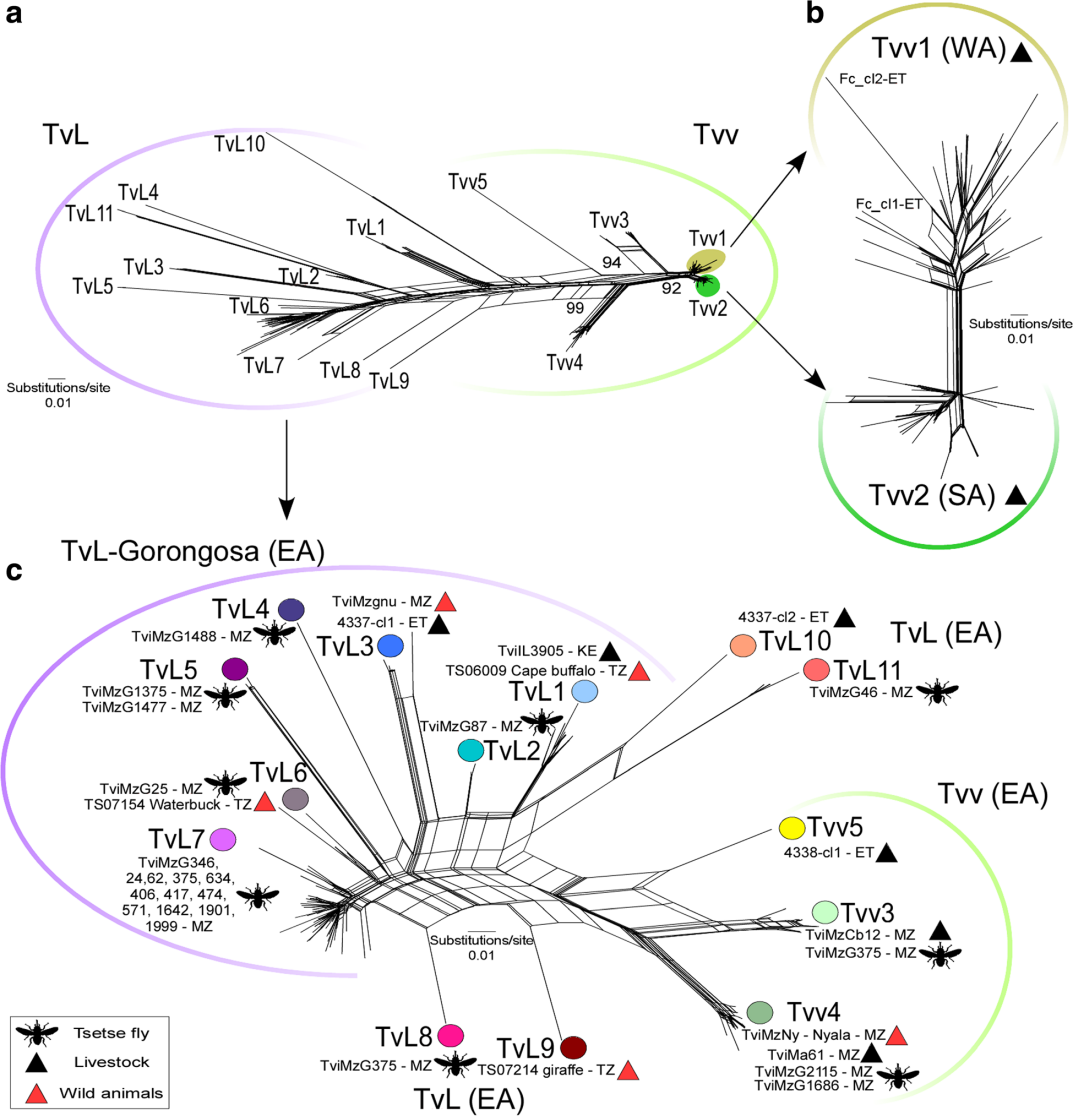
Network split decomposition of ITS rDNA sequences from *T. vivax* and *T. vivax*-like, inferred using the Neighbour-Net method. **a** Analysis of 263 ITS rDNA sequences (87 samples) from South America (SA), West Africa (WA) and East Africa (EA). **b** Network restricted to dataset from closely related isolates from SA, WA and Ethiopia supporting the separation between SA and WA, as well as greater heterogeneity of WA compared to SA isolates, and relevant divergence of Ethiopian sequences. **c** Network restricted to EA dataset supporting TvL1-TvL7 genotypes of the lineage TvL-Gorongosa, and divergent TvL8-TvL11 likely representing additional TvL lineages

To investigate both the relationships and the possibility of recombination generating the remarkable polymorphic ITS sequences, we submitted the dataset to network split decomposition. All sequences from WA and SA clustered tightly together, whereas EA sequences largely dispersed in a complex and reticulated network (Fig. 5a).

The split between SA and WA sequences corroborated the MDS results and showed more relevant polymorphisms in Tvv1 compared to Tvv2 (Fig. 5b). Each isolate from WA exhibited a unique ITS sequence. Ethiopian isolates were assigned to Tvv1 and Tvv5, which are closely related to Tvv3 and Tvv4 from MZ, and to Tvv2 from SA (Fig. 5c).

Consistent with MDS analysis (Fig. 4), split decomposition network of ITS sequences uncovered greater diversity within TvL compared to Tvv. MDS clustering, network patterns, and polymorphisms on the aligned ITS sequences (Additional file 3: Figure S1) were all consistent with the lineage TvL-Gorongosa and its genotypes (Fig. 5c).

There was a high concordance between groups/genotypes defined by gGAPDH and ITS rDNA (Table 1). Genotypes TvL1 and TvL2 include isolates from tsetse, livestock and wild animals from MZ, whereas TvL3 clustered isolates from Kenyan cattle and Tanzanian buffalo. TvL1-TvL3 belongs to TvLA/B assemblage defined by gGAPDH, and TvL4-TvL7 are likely of TvLC/D grouping. Taking into account their close relationships and small degrees of sequence divergences, TvL1–7 are all considered genotypes of TvL-Gorongosa. This lineage comprises most ITS sequences obtained from tsetse and from wild ungulates from MZ and Tanzania, in addition to cattle isolates from Kenya and Ethiopia (Fig. 5c). Long branches in the network correspond to EA sequences assigned to TvL8–11 genotypes.

Results from our comprehensive analysis corroborate the high discriminatory power of ITS rDNA sequences allowing for the identification of known and novel genotypes. However, deeper nodes within Tvv and TvL remain uncertain. The ITS network displayed noticeable reticulation of TvL sequences and a moderate degree of reticulation among Tvv genotypes from EA, suggesting that EA populations may undergo genetic recombination in tsetse flies, whereas the small degree of reticulation among Tvv sequences from WA and SA suggest clonal populations.

## Discussion

### *Trypanosoma vivax* and *Trypanosoma vivax-*like in tsetse flies and wild reservoirs from Mozambique

This is the first molecular survey of *T. vivax* in tsetse flies from MZ. *Glossina m. morsitans* was the predominant species, found in sympatry with a smaller population of *G. pallidipes* in GNP and NNR. These tsetse species are highly effective vectors of trypanosomes that are pathogenic to humans and livestock in the great Zambezi Valley and across EA [35–37]. Despite the absence of large-scale surveys on tsetse flies and animal trypanosomiasis in MZ, *Glossina m. morsitans* and *G. pallidipes* are known to be present from northern to central MZ. The investigations carried out in central provinces, including Sofala where GNP is situated, have shown that due to a high prevalence of Nagana, cattle production throughout large areas of Mozambique has to rely on preventive treatment against trypanosomiasis [38]. The present study aimed to characterise *T. vivax* isolates; a more substantial understanding of tsetse diversity and prevalence in MZ is beyond the scope of this work.

We investigated the presence of *T. vivax* in tsetse flies from GNP and NNR using the FFLB method [30, 39] previously employed for surveys of trypanosomes in tsetse flies from the wildlife reserves of Tarangire, Serengeti and Msubugwe in Tanzania [26, 29]. FFLB detected *T. vivax* and/or *T. vivax*-like in 25.6% of flies examined. The method of TviCATL-PCR [20] was herein confirmed as a simple, sensitive and specific diagnostic method, regardless of the parasite lineage/genotype or the existence of multiple trypanosome species. Despite being limited to a few samples, molecular surveys have identified *T. vivax* in a range of wild ungulates including antelopes, Cape buffalo, warthog and giraffe in Tanzania, Zambia and MZ [2, 24, 40, 41]. These findings corroborated morphological studies of blood trypanosomes reporting *T. vivax* and *T. vivax*-like in a range of wild ungulates in EA [42, 43].

### *Duttonella* comprises *T. vivax* and *T. vivax-*like phylogenetic lineages constituted by a greater genotype repertoire in East Africa compared with West Africa and South America

There are growing amounts of molecular data uncovering a great repertoire of trypanosomes including new species and genotypes infecting tsetse flies in wildlife reserves. Novel trypanosomes from tsetse flies [7, 25, 26, 29, 30, 44–46] and African ungulates [2, 23, 24, 34] were reported in eastern and central-southern African wildlife conservation areas.

Here, inferences based on gGAPDH sequences strongly supported two major evolutionary lineages, Tvv and TvL, each one composed of four main groups. Relevant congruence among lineages/groups defined by gGAPDH and ITS rDNA sequences were demonstrated by MDS, phylogenetic trees and network approaches. Genotyping within each evolutionary lineage was better assessed using highly discriminatory ITS sequences. Tvv lineage includes the reference strain *T. vivax* Y486 of cattle from Nigeria [47]. This lineage comprises genotypes Tvv1, including all WA plus some EA sequences, Tvv2, exclusively from SA, and Tvv3-Tvv5, so far restricted to some EA samples. All analyses strongly supported a remarkable diversity within TvL formed by at least 11 genotypes represented, so far, by sequences from MZ, Tanzania, Kenya and Ethiopia [2, 27]. Most TvL genotypes were identified in tsetse flies, four were detected in wild ungulates, and only three genotypes were identified in livestock. Genotypes uncovered by our study almost certainly represented only a small part of the genetic diversity within the *Duttonella* subgenus.

Taking into account the remarkable diversity of *Duttonella,* and the intertwined network branching patterns of ITS rDNA sequences, it is tempting to speculate that in EA, new genotypes may have emerged by some recombination process during the parasite development in tsetse flies. Studies supporting clonal propagation of *T. vivax* in WA and SA populations have demonstrated the stability of predominant genotypes over time consistent with clonality [8, 22]. However, all data from WA and SA came from livestock production zones free of tsetse (SA) or with smaller tsetse fly density compared to GNP and NNR.

### The subgenus *Duttonella* is a complex of species and genotypes

The gGAPDH phylogenetic inference strongly supports one monophyletic assemblage harbouring all isolates from tsetse flies and ungulates identified as *T. vivax* or *T. vivax-*like corresponding to the subgenus *Duttonella*. Phylogenetic inferences support *Duttonella* as the most basal clade*,* corroborating previous study results, indicating that it was the first lineage to diverge from the common ancestor of the phylogenetic clade *T. brucei,* which comprises the subgenera *Duttonella*, *Nannomonas*, *Trypanozoon* and *Pycnomonas* [19, 24, 48]. Representing the early branching African trypanosome, *T. vivax* has been explored by whole genomic and transcriptomic studies, showing significant differences from other African trypanosomes in the cell-surface developmentally regulated proteins and mitochondrial genomes, suggesting significant peculiarities in the parasite interaction with its ungulate hosts and vectors [49–51].

Phylogenetic positioning and sequence divergence separating *T. vivax* Y486 from TvL-Gorongosa justified its identification as another species of *Duttonella*. Our findings greatly expanded the species/genotype richness that has been discovered by phylogenetic analyses within the subgenus *Duttonella*. The possibility that genetic recombination has shaped the high genetic diversity observed in EA should be specifically addressed in further microsatellite, multilocus and whole genome studies. This is a very relevant question since the creation of genetic variants may produce undesirable parasite features related to pathogenicity, virulence and drug resistance, as well as being a source of novel genotypes for outbreaks [52].

### *Trypanosoma vivax* and other species of *Duttonella* most likely arose in East Africa, from where genotypes adapted to livestock may have spread across Africa and South America

The greater diversity in EA conservation areas of trypanosome genotypes either highly similar or divergent compared with those from WA, and the basal position of TvL in phylogenetic trees, suggest that EA was the region of origin and diversification of *Duttonella.* Possibly, a range of genotypes emerged through recombination of genetically different parasites circulating between wild tsetse and several species of ungulates. Some genotypes may have adapted to livestock, then spread across EA and WA with the historical movement of infected livestock, accompanying human migration along sub-Saharan Africa. In Africa, *T. vivax* has a wide range of healthy domestic reservoirs, including native breeds of cattle, goats, sheep, donkeys, camels and even suids. Both migratory routes from WA to EA and vice versa may have played some role in the spreading of Tvv1 in cattle from The Gambia to Ethiopia. In MZ, we identified cattle infected with Tvv3 and Tvv4 genotypes that are highly closely related to Tvv1, but the existence of Tvv1 in this country, as well as in Tanzania and Kenya, must be assessed by examining more comprehensive cattle sampling.

In EA, cattle were found to be infected by Tvv and TvL genotypes as confirmed here in MZ and reported previously in Ethiopia, as well as in Zambia, Central-Southern Africa [7, 23, 25–27]. Despite the small sample, Ethiopian isolates clustered either into Tvv or TvL. The observation that some *T. vivax* genotypes from Ethiopian are closely related to WA genotypes [7] agreed with previous reports on isolates from MZ, which were different, but more related to those from WA than to those from EA (Tanzania and Kenya) [20, 24–26]. In addition to Ethiopia, Tvv genotypes closely related to those from WA/SA has been found in Zambia [23] and Uganda (Garcia et al. in preparation). Thus far, Tvv1 was the only one that has been found both in WA and EA, being highly prevalent in WA where the existence of other genotypes needs to be investigated. However, different from highly homogeneous SA populations of Tvv2 and despite tightly clustered together, substantial genetic diversity has been revealed among Tvv1 isolates, as demonstrated in this and in previous studies of isolates from The Gambia, Burkina Faso, Benin, Ghana, Nigeria and Cameroon [4, 8, 23, 24].

This study corroborated a tight relationship between Tvv1 (WA) and Tvv2 (SA) genotypes. However, to date, not a single ITS rDNA sequence from Africa was identical to those of Tvv2, which comprises all the 39 samples from Brazil, Venezuela, Colombia and French Guiana examined in our study. Our findings support the hypothesis that Tvv2 may be a bottlenecked genotype that recently diverged from closely related genotypes escaped out of WA. In a study based on microsatellite markers, we also demonstrated that *T. vivax* from WA and SA are highly similar, but not identical, and that diversity was far greater across WA than SA [8].

To date, all TvL isolates came from tsetse-infested areas in EA. Although an early study carried out in Ethiopia suggested that isolates from tsetse-free areas clustered with SA/WA genotypes, while those from tsetse-infested areas clustered with EA genotypes, analysis based on polymorphic ITS sequences did not support this hypothesis [7, 27]. In Ethiopia and other African countries, *T. vivax* is cyclically transmitted by tsetse flies and mechanically transmitted by other biting flies [6, 7, 27]. In SA, *T. vivax* is only mechanically transmitted by non-tsetse biting flies. Genomic studies comparing Venezuelan and WA *T. vivax* Y486 evidenced a drastic process of mitochondrial genome degradation in SA isolates, whereas the African *T. vivax* Y486 exhibited entirely functional mitochondria necessary for the development in tsetse flies [51].

### Taxonomy and morphological, biological and immunopathological peculiarities of *Duttonella* trypanosomes in East Africa

Our study uncovered a complex of species/genotypes within the subgenus *Duttonella.* Molecular data provided herein corroborate differences between WA and EA *T. vivax* and among EA isolates regarding their morphology, infectivity to tsetse flies, pathogenicity and antigenic relationships. In the last taxonomical revision of *Trypanosoma* [42], in addition to *T.* (*Duttonella*) *vivax vivax* (= *T. vivax*) that is the type-species of the subgenus [42], *T.* (*Duttonella*) *uniforme*, was recognised as a valid species of *Duttonella,* characterised by small-sized blood forms. This species was reported in cattle, antelope, buffalo and giraffe, widespread in Uganda and Congo, and reported in Zululand, Tanzania and Ethiopia. *T.* (*Duttonella*) *vivax ellipsiprymni* was regarded as a subspecies due to the morphological intergradation of its blood forms with those of *T. vivax* [42, 53]. *Trypanosoma uniforme* and *T. v. ellipsiprymni* were thought to be transmitted by *G. morsitans*, *G. palpalis* and *G. fuscipes* in EA [42]. It is possible that these trypanosomes correspond to *T. vivax*-like reported in this and in previous studies [2, 7, 19, 23–26]. We previously described a *T. vivax-*like from a nyala antelope in MZ showing large blood trypomastigotes morphologically resembling those of *T. v. ellipsiprymni* [24]. This isolate was herein assigned to Tvv, and differ only by 0.7% of gGAPDH sequence divergence from *T. vivax* Y486.

Recently, *T.* (*Pycnomonas*) *suis*, which was initially phylogenetically positioned near *T. brucei* and provisionally named *Trypanosoma* sp. Musubugwe, was molecularly validated using DNA recovered from archived blood smears [46]. Isolates cryopreserved and archived collections of tsetse flies and ungulate blood smears of species of *Duttonella* can serve as DNA source for comparison with species candidates uncovered in this and other studies.

We identified different genotypes of Tvv and TvL in *G. m. morsitans* and *G. pallidipes*. Probably because *G. morsitans* was highly predominant in our study, a larger repertoire of trypanosomes was found in this species*.* Trypanosomes from Tanzanian *G. pallidipes* and *G. swynnertoni* [25, 26] slightly differed from those we identified in MZ tsetse flies. In accordance with long-range distribution, it was demonstrated that WA *T. vivax* developed in a range of *Glossina* species. In contrast, EA isolates of *Duttonella* likely prefer sympatric tsetse flies. Nevertheless, *G. pallidipes* was shown to be equally competent in experimentally transmitting *T. vivax* from Kenya and Nigeria [54, 55]. The presence of both TvL and Tvv in EA countries may be related to the coexistence of a range of tsetse species [56] feeding on many species of wild animals and large herds of livestock.

Corroborating the close relationships between *T. vivax* populations in WA, high antigenic cross-reactivity was reported for isolates from The Gambia and Nigeria, but not between these and distantly related Kenyan isolates. In addition, isolates from Kenya differed in resistance to drugs [57], antigenic cross-reactivity and pathogenicity. The severe hemorrhagic syndrome has, so far, been reported in Kenya and Uganda [10, 11]. Studies reporting a lack of significant differences in pathogenicity of *T. vivax* isolates from areas that were or were not infested by tsetse flies in Ethiopia did not include molecular characterization of the isolates [6]. Despite considerable immunopathological data on *T. vivax* infected cattle in Kenya, Ethiopia and Uganda, genetic diversity of a comprehensive sampling remains to be investigated.

## Conclusions

Our comparative study of a large sample of *T. vivax* and *T. vivax*-like gGAPDH and ITS rDNA sequences uncovered two major evolutionary lineages, constituted by several genotypes, within the subgenus *Duttonella*: *T. vivax* (Tvv) composed of five genotypes, and *T. vivax*-like (TvL) constituted by TvL-Gorongosa, an assemblage of seven genotypes, in addition to genotypes that likely belong to other TvL lineages*.* Phylogenetic inference supports the rising of TvL-Gorongosa to the species status. We detected TvL-Gorongosa in *G. m. morsitans* and *G. pallidipes*, as well as in wild and domestic ungulates, in MZ, Tanzania, Kenya and Ethiopia. However, before proposing any new species attempts to compare this new lineage with previously reported species and subspecies of *Duttonella* [42] are highly recommended. Our analyses point to many novel species/subspecies and genotypes remaining to be discovered in this subgenus. In addition, EA isolates must be genotyped and evaluated in experimental infections regarding differential behaviour, clinical signs, and pathological and immunological aspects. Data generated by comparison of phenotypic and genotypic (including multilocus and whole genome studies) characteristic are badly required to improve our understanding about *Duttonella* trypanosomes. Genetic diversity was noticeable in EA wildlife conservation areas, where wild ungulates serve as reservoirs for a large diversity of Tvv and TvL genotypes that are transmitted by a range of tsetse species*.* Not a single TvL isolate was identified in WA, where *T. vivax* population shown to be quite homogeneous, even though samples examined came from The Gambia to Cameroon, over several years. To date, a single genotype was common in livestock from WA and EA. Further studies on the genetic diversity of *T. vivax* in wild tsetse and ungulates in WA are required to evaluate whether the lower diversity found in WA was because all isolates examined were obtained from cattle. Data from this study provide additional support for the hypothesised bottlenecked SA population, recently imported from WA. The geographical origin of any pathogenic trypanosome is an issue of high interest, and information on the genetic diversity is fundamental to understand its distribution. We suggested an EA origin for *Duttonella* species*,* and hypothesised the spread of Tvv genotypes adapted to livestock, and transmitted either cyclically by tsetse or mechanically by other flies, following historical livestock dispersion routes. Any data on the genetic and spatial structure is valuable for assessing possible links between genotypes and vertebrate hosts, vector species, pathogenicity, and drug resistance of *T. vivax* and *T. vivax-*like.

## Additional files

Additional file 1: Table S1. *Trypanosoma vivax* isolates from Africa, including the host species, geographical origin and groups/genotypes defined by gGAPDH and ITS rDNA analyses. TvL-G: TvL-Gorongosa. (DOCX 29 kb)

Additional file 2: Table S2. *Trypanosoma vivax* isolates from South America, including the host species, geographical origin and groups/genotypes defined by gGAPDH and ITS rDNA analyses. (DOCX 19 kb)

Additional file 3: Figure S1. The alignment of ITS1 and ITS2 rDNA sequences from *Trypanosoma vivax* and *T. vivax*-like isolates. South American and West African isolates shared highly conserved sequences, exhibiting only punctual polymorphisms. The polymorphic East African isolates revealed blocks of nucleotides that were unique for each genotype, as well as conserved segments shared by closely related genotypes. The reference isolate/sequence representing each genotype is underlined. ITS sequences were deposited in GenBank (accession numbers in Additional files 1 and 2). (TIFF 496 kb)

## Abbreviations

EA: East Africa
FFLB: fluorescent fragment length barcoding
gGAPDH: glycosomal glyceraldehyde phosphate dehydrogenase
GNP: Gorongosa National Park
ITS rDNA: internal transcribed spacer of rDNA
MZ: Mozambique
NNR: Niassa National Reserve
SA: South America
TviCATL-PCR: *T. vivax*-specific PCR assay based on cathepsin L gene
Tvv: *T. vivax* lineage TvL *T. vivax*-like lineage
WA: West Africa
