# Supplementary material for: New insights from Gorongosa National Park and Niassa National Reserve of Mozambique increasing the genetic diversity of Trypanosoma vivax and Trypanosoma vivax-like in tsetse flies, wild ungulates and livestock from East Africa
Source: Parasit Vectors. 2017 Jul 17;10:337. doi: 10.1186/s13071-017-2241-2 (PMC5513381; doi:10.1186/s13071-017-2241-2)
Supplement: Supplementary file 1 — Trypanosoma vivax isolates from Africa, including the host species, geographical origin and groups/genotypes defined by gGAPDH and ITS rDNA analyses. TvL-G: TvL-Gorongosa. (DOCX 29 kb) [file 13071_2017_2241_MOESM2_ESM.docx]

**Additional File 1**

**Table A1. *Trypanosoma vivax* isolates from Africa, host species, geographic origin and genotypes defined by gGAPDH and ITS rDNA sequences**

| *T. vivax*  isolates | gGAPDH group | | ITS  genotype | | Host  species | | Geographic  origin | Date | GenBank  gGAPDH | | GenBank  ITS rDNA | | References |
| --- | --- | --- | --- | --- | --- | --- | --- | --- | --- | --- | --- | --- | --- |
| Y486 ^EX^ | TvvA (1/1) | Tvv1 (1/1) | | cattle | | | Nigeria | 1976 | | - | | U22316 | 47 |
| TviBfMatorkou | TvvA (2/2) | Tvv1 (4/4) | | cattle | | | Burkina Faso | 2008 | | KX584751 | | KC196673-KC196676 | 8 |
| TviDere091 | TvvA (3/3) | Tvv1 (4/4) | | cattle | | | Ghana | 2008 | | KX584760 | | KC196638-KC196641 | 8 |
| TviBan1 | TvvA (3/3) | Tvv1 (3/3) | | cattle | | | Benin | 2008 | | KX584753 | | KC196648-KC196650 | 8 |
| Gambia ^EX^ | TvvA (1/1) | Tvv1 (3/3) | | cattle | | | The Gambia | 2009 | | KX584752 | | KC196658-KC196660 | 8 |
| FP9 | TvvA (1/1) | Nd | | cattle | | | Cameroon | - | | FN400713 | | - | 25 |
| FN400714 | TvvA (1/1) | Nd | | cattle | | | Gambia | - | | FN400714 | | - | 25 |
| TviMzCb11 | TvvB (2/2) | Nd | | cattle | | | Mozambique^T^ | 2007 | | KX584761- KX584762 | | - | This study |
| TviMzSoAbu21 | TvvB (1/10),  TvLB (8/10),  TvLC (1/10) | Nd | | buffalo | | | Mozambique^So^ | 2011 | | KX584763  KX584784  KX584789- KX584793  KX584797 | | - | This study |
| TviMzG545 | TvvB (1/1) | Nd | | tsetse^#^ | | | Mozambique^N^ | 2014 | | KX584767 | | - | This study |
| TviKC92J28 | TvvB (3/3) | Tvv1 (4/4) | | cattle | | | Ghana | 2008 | | KX584756 | | KC196630-KC196633 | 8 |
| TviKang92 | TvvB (3/3) | Tvv1 (6/6) | | cattle | | | Ghana | 2008 | | KX584759 | | KC196620-KC196625 | 8 |
| Desowitz | TvvB (1/1) | Nd | | sheep | | | Nigeria | 2005 | | AJ620295 | | - | 30 |
| AF047500 | TvvB (1/1) | Tvv1 | | - | | | - | - | | AF047500 | | - | 59 |
| TviMzG68 | TvvC (2/3)  IG (1/3) | Nd | | tsetse^#^ | | | Mozambique^G^ | 2012 | | KX584772  KX584774  KX584835 | | - | This study |
| TviBfL44 5 | nd | Tvv1 (4/4) | | cattle | | | Burkina Faso | 2008 | | - | | KC196669-KC196672 | 8 |
| TviBfFolonzo | nd | Tvv1 (2/2) | | cattle | | | Burkina Faso | 2008 | | - | | KC196677-KC196678 | 25 |
| TviBfMene | nd | Tvv1 (8/8) | | cattle | | | Burkina Faso | 2008 | | - | | KC196661-KC196668 | 8 |
| TviKCA19J56 | nd | Tvv1 (8/8) | | cattle | | | Ghana | 2008 | | - | | KC196634-KC196637 | 8 |
| TviBan1.2 | nd | Tvv1 (3/3) | | cattle | | | Benin | 2008 | | - | | KC196651-KC196653 | 8 |
| TviKommon | nd | Tvv1 (4/4) | | cattle | | | Benin | 2008 | | - | | KC196642-KC196645 | This study |
| TviBolonsi063 | nd | Tvv1 (2/2) | | cattle | | | Benin | 2008 | | - | | KC196646-KC196647 | This study |
| IL700 | nd | Tvv1 (4/4) | | cattle | | | Nigeria | - | | - | | KC196654-KC196657 | 25 |
| TviBfL44 5 | nd | Tvv1 (4/4) | | cattle | | | Burkina Faso | 2008 | | - | | KC196669-KC196672 | 8 |
| TviMzG375 | TvvC (1/1) | Tvv3 (1/3),  TvL-G7(1/3),  TvL8 (1/3) | | tsetse^ni^ | | | Mozambique^G^ | 2007 | | KX584783 | | KX584842- KX584843  KX584884 | This study |
| TviMzG1926 | TvvC (1/1) | Nd | | tsetse^#^ | | | Mozambique^G^ | 2009 | | KX584775 | | - | This study |
| TviMzG2172 | TvvC (4/4) | Nd | | tsetse^#^ | | | Mozambique^G^ | 2009 | | KX584779 | | - | This study |
| TviMzG2175 | TvvC (1/1) | Nd | | tsetse^ni^ | | | Mozambique^G^ | 2009 | | KX584780 | | - | This study |
| TviMzG2194 | TvvC (1/1) | Nd | | tsetse^■^ | | | Mozambique^G^ | 2009 | | KX584781 | | - | This study |
| TviMzG215 | TvvC (5/5) | Nd | | tsetse^#^ | | | Mozambique^N^ | 2013 | | KX584776  KX584777 | | - | This study |
| TviMzG403 | TvvC (2/4),  TvLC (2/4) | Nd | | tsetse^#^ | | | Mozambique^N^ | 2014 | | KX584782  KX584794  KX584804 | | - | This study |
| TviMzG405 | TvvC (5/5) | Nd | | tsetse^#^ | | mozambique^n^ | | 2014 | | KX584778 | | - | This study |
| TviMzG449 | TvvC (3/3) | Nd | | tsetse^#^ | | mozambique^n^ | | 2014 | | KX584771  KX584773 | | - | This study |
| TviMzNy | TvvD (1/1) | Tvv4 (5/5) | | nyala | | mozambique^so^ | | 2006 | | FM876218 | | EU482078–EU482082 | 24 |
| TviMzCb12 | TvvD (1/1) | Tvv3 (9/9) | | cattle | | mozambique^t^ | | 2007 | | FM876219 | | KC196679-KC196687 | 20 |
| TviMzG1686 | TvvD (3/4)  IG (1/4) | Tvv4 (7/7) | | tsetse^#^ | | mozambique^g^ | | 2009 | | KX584770  KX584837 | | KX584844 | This study |
| TviMzG2115 | TvvD (2/13)  TvLC (10/13)  TvLD (1/13) | Tvv4 (1/1) | | tsetse^#^ | | mozambique^g^ | | 2009 | | KX584769  KX584821  KX584822  KX584831 | | KC196699 | This study |
| TviMzG464 | TvvD (1/1) | Nd | | tsetse^#^ | | mozambique^n^ | | 2014 | | KX584768 | | - | This study |
| FM164787 | TvLA (1/1) | Nd | | tsetse* | | tanzania^s^ | | 2006/7 | | FM164787 | | - | 25 |
| FM164788 | TvLA (1/1) | Nd | | tsetse* | | tanzania^s^ | | 2006/7 | | FM164788 | | - | 25 |
| TviIL3905 | TvLA (3/3) | TvL-G1 (9/9) | | cattle | | kenya | | 1986 | | KX584747 | | DQ316039–DQ316044 | 24 |
| FM164786 | TvLA (1/1) | Nd | | tsetse* | | tanzania^s^ | | 2006/07 | | FM164786 | | - | 25 |
| FM164789 | TvLA (1/1) | Nd | | tsetse* | | tanzania^s^ | | 2006/07 | | FM164789 | | - | 25 |
| TviMzCb3 | TvLB (2/2) | Nd | | cattle | | mozambique^t^ | | 2007 | | KX584787  KX584788 | | - | This study |
| TviMzGnu12 | TvLB (2/2) | TvL-G3 (6/6) | | gnu | | mozambique^n^ | | 2013 | | KX584785 | | KX584883 | This study |
| TviMzG46 | TvLB (2/2) | Nd | | tsetse^ni^ | | mozambique^g^ | | 2007 | | KX584786 | | - | This study |
| TviMzG404 | TvLC (5/5) | Nd | | tsetse^#^ | | mozambique^n^ | | 2014 | | KX584801  KX584811 | | - | This study |
| TviMzG433 | TvLC (3/3) | Nd | | tsetse^#^ | | mozambique^n^ | | 2014 | | KX584800  KX584812 | | - | This study |
| TviMzG474 | TvLC (2/2) | TvL-G7 (4/4) | | tsetse^ni^ | | mozambique^g^ | | 2007 | | KX584813  KX584815 | | - | This study |
| TviMzG510 | TvLC (2/2) | Nd | | tsetse^#^ | | mozambique^n^ | | 2014 | | KX584795 | | - | This study |
| TviMzG571 | TvLC (4/7), IG(3/7) | TvL-G7 (8/8) | | tsetse^ni^ | | mozambique^g^ | | 2007 | | KX584796  KX584799  KX584814  KX584836  KX584838  KX584839 | | KX584851- KX584856  KX584872- KX584873  KX584875 | This study |
| TviMzG647 | TvLC (2/2) | Nd | | tsetse^#^ | | mozambique^g^ | | 2009 | | KX584798 | | - | This study |
| TviMzG1477 | TvLC (1/1) | TvL-G5 (1/1) | | tsetse^#^ | | mozambique^g^ | | 2009 | | KX584802 | | KX584881 | This study |
| TviMzG1488 | TvLC (4/4) | TvL-G4 (1/1) | | tsetse^#^ | | mozambique^g^ | | 2009 | | KX584803  KX584805  KX584806 | | KX584882 | This study |
| TviMzG1585 | TvLC (1/1) | Nd | | tsetse^■^ | | mozambique^g^ | | 2009 | | KX584807 | | - | This study |
| TviMzG1642 | TvLC (2/2) | TvL-G7 (5/5) | | tsetse^#^ | | mozambique^g^ | | 2009 | | KX584808  KX584809 | | KX584857- KX584860  KX584866 | This study |
| TviMzG1860 | TvLC (2/2) | Nd | | tsetse^#^ | | mozambique^g^ | | 2009 | | KX584816  KX584834 | | - | This study |
| TviMzG1901 | TvLC (5/5) | TvL-G7 (6/6) | | tsetse^#^ | | mozambique^g^ | | 2009 | | KX584810  KX584817 | | KX584861- KX584865  KX584874 | This study |
| TviMzG1951 | TvLC (1/1) | Nd | | tsetse^■^ | | mozambique^g^ | | 2009 | | KX584818 | | - | This study |
| TviMzG719 | TvLC (1/1) | Nd | | tsetse^#^ | | mozambique^g^ | | 2009 | | KX584819 | | - | This study |
| TviMzG417 | TvLD (1/1) | TvL-G7 (1/1) | | tsetse^ni^ | | mozambique^g^ | | 2007 | | KX584829 | | KX584846 - KX584850 | This study |
| TviMzG634 | TvLD (1/1) | TvL-G7 (2/2) | | tsetse^#^ | | mozambique^g^ | | 2009 | | KX584825 | | KC196700-KC196701 | This study |
| TviMzG1046 | TvLD (10/10) | Nd | | tsetse^■^ | | mozambique^g^ | | 2009 | | KX584820  KX584823  KX584824  KX584826-  KX584828 | | - | This study |
| TviMzG10 | TvLD (1/3)  IG (2/3) | Nd | | tsetse^#^ | | mozambique^g^ | | 2012 | | KX584830  KX584840  KX584841 | | - | This study |
| TviMzG24 | TvLD (2/2) | TvL-G7 (2/2) | | tsetse^#^ | | mozambique^g^ | | 2012 | | KX584832-  KX584833 | | KX584870- KX584871 | This study |
| FM164790 | IG(1/1) | Nd | | tsetse* | | tanzania^s^ | | 2006/7 | | FM164790 | | - | 25 |
| TviMzG87 | Nd | TvL-G2 (4/4) | | tsetse^ni^ | | mozambique^g^ | | 2007 | | - | | KC196688-KC196691 | This study |
| TviMzMa61 | Nd | Tvv4 (5/5) | | cattle | | mozambique^m^ | | 2007 | | - | | KX584845 | 20 |
| TviMzG1375 | Nd | TvL-G5 (4/4) | | tsetse^#^ | | mozambique^g^ | | 2009 | | - | | KX584880 | This study |
| TviMzG25 | Nd | TvL-G6 (2/2) | | tsetse^ni^ | | mozambique^g^ | | 2012 | | - | | KX584876- KX584877 | This study |
| TviMzG346 | Nd | TvL-G7 (4/4) | | tsetse^#^ | | mozambique^g^ | | 2007 | | - | | KC196692-KC196695 | This study |
| TviMzG406 | Nd | TvL-G7 (3/3) | | tsetse^#^ | | mozambique^g^ | | 2007 | | - | | KC196696-KC196698 | This study |
| TviMzG1999 | Nd | TvL-G7 (2/2) | | tsetse^#^ | | mozambique^g^ | | 2009 | | - | | KC196702-KC196703 | This study |
| TviMzG62 | Nd | TvL-G7 (3/3) | | tsetse^#^ | | mozambique^g^ | | 2012 | | - | | KX584867- KX584869 | This study |
| TviMzG46 | Nd | TvL11 (2/2) | | tsetse^ni^ | | mozambique^g^ | | 2012 | | - | | KX584878- KX584879 | This study |
| TS06009-TZ | Nd | TvL-G1 (1/1) | | buffalo | | tanzania^s^ | | 2006 | | - | | JN673394 | 2 |
| TS07214-TZ | Nd | TvL9 (1/1) | | giraffe | | tanzania^s^ | | 2007 | | - | | JN673392 | 2 |
| TS07154-TZ | Nd | TvL-G6 (1/1) | | waterbuck | | tanzania^s^ | | 2007 | | - | | JN673393 | 2 |
| 4337-ET | nd | TvL-G3 (1/2)  TvL10 (1/2) | | cattle | | ethiopia^j^ | | 2012 | | - | | KM391820-KM391830  KM391821-KM391831 | 27 |
| 4338-ET | nd | Tvv5 (1/1) | | cattle | | ethiopia^j^ | | 2012 | | - | | KM391822-KM391832 | 27 |
| Fc-ET | nd | Tvv1 (2/2) | | cattle | | ethiopia^b^ | | 2012 | | - | | KM391826-KM391834  KM391827-KM391835 | 27 |

Tvv = Lineage *T. vivax* ; TvL = Lineage *T. vivax*-like; TvL-G = Lineage TvL-Gorongosa. #, *Glossina morsitans*; *, *Glossina swynnertoni*; ■, *Glossina pallidipes*; Mozambique: M, Matutuíne, T, Tete; G, Gorongosa; N, Niassa; So, Sofala. Tanzania: S, Serengueti; Ethiopia: J, Jimma zone, B, Bale Zone. Nd, Not determined, IG, indeterminate genotype, NI, Not identified.
