# Supplementary material for: New insights from Gorongosa National Park and Niassa National Reserve of Mozambique increasing the genetic diversity of Trypanosoma vivax and Trypanosoma vivax-like in tsetse flies, wild ungulates and livestock from East Africa
Source: Parasit Vectors. 2017 Jul 17;10:337. doi: 10.1186/s13071-017-2241-2 (PMC5513381; doi:10.1186/s13071-017-2241-2)
Supplement: Supplementary file 2 — Trypanosoma vivax isolates from South America, including the host species, geographical origin and groups/genotypes defined by gGAPDH and ITS rDNA analyses. (DOCX 19 kb) [file 13071_2017_2241_MOESM3_ESM.docx]

**Additional File 3:**

**Table A2.** *Trypanosoma vivax* isolates from South America, host and geographic origin, and gGAPDH groups and ITS rDNA genotypes

| ***T. vivax***  **isolates** | **gGAPDH genotype** | **ITS**  **genotype** | **Host**  **species** | **Geographic**  **origin** | **Date of**  **isolation** | **GenBank**  **gGAPDH** | **GenBank**  **ITS rDNA** | **References** |
| --- | --- | --- | --- | --- | --- | --- | --- | --- |
| TviBrPA14 | TvvB (2/2) | Tvv2 (1/1) | buffalo | Brazil, PA | 2008 | KX584758 | KC196575 | 8 |
| TviBrPA29 | Nd | Tvv2 (1/1) | buffalo | Brazil, PA | 2008 | - | KC196576 | 8 |
| TviBrPA32 | Nd | Tvv2 (2/2) | buffalo | Brazil, PA | 2008 | - | KC196577-KC196578 | 8 |
| TviBrPA41 | Nd | Tvv2 (2/2) | buffalo | Brazil, PA | 2008 | - | KC196559-KC196560 | 8 |
| TviBrPA45 | Nd | Tvv2 (2/2) | buffalo | Brazil, PA | 2008 | - | KC196561-KC196562 | 8 |
| TviBrPA49 | Nd | Tvv2 (2/2) | cattle | Brazil, PA | 2008 | - | KC196555-KC196556 | 8 |
| TviBrPA50 | Nd | Tvv2 (2/2) | cattle | Brazil, PA | 2008 | - | KC196557-KC196558 | 8 |
| TviBrPA52 | Nd | Tvv2 (4/4) | cattle | Brazil, PA | 2007 | - | KC196544-KC196547 | 8 |
| TviBrSP2 | TvvB (1/1) | Tvv2 (1/1) | cattle | Brazil, SP | 2008 | KX584755 | KC196563 | 8 |
| TviBrCa | TvvA (1/1) | Nd | cattle | Brazil, PB | 2002 | FM876221 | FM876221 | 8 |
| TviBrRp | TvvB (4/4) | Nd | sheep | Brazil, PB | 2008 | KX584764- KX584765 | - | 15 |
| TviBrPB27 | Nd | Tvv2 (2/2) | sheep | Brazil, PB | 2008 | - | KC196548-KC196549 | 15 |
| TviBrPB28 | Nd | Tvv2 (2/2) | sheep | Brazil, PB | 2008 | - | KC196550-KC196551 | 15 |
| TviBrPB30 | Nd | Tvv2 (3/3) | sheep | Brazil, PB | 2008 | - | KC196552-KC196554 | 15 |
| TviBrPB50 | Nd | Tvv2 (3/3) | sheep | Brazil, PB | 2009 | - | KC196566-KC196568 | 15 |
| TviBrPB52 | Nd | Tvv2 (2/2) | sheep | Brazil, PB | 2009 | - | KC196573-KC196574 | 8 |
| TviBrPB51 | Nd | Tvv2 (2/2) | buffalo | Brazil, PB | 2009 | - | KC196569-KC196570 | 15 |
| TviBrPB53 | Nd | Tvv2 (2/2) | buffalo | Brazil, PB | 2009 | - | KC196571-KC196572 | 8 |
| TviBrBov1 | Nd | Tvv2 (1/1) | cattle | Brazil, MS | 2000 | - | - | 8 |
| TviBrPo | Nd | Tvv2 (2/2) | cattle | Brazil, MS | 1996 | - | DQ316049, DQ316050 | 19 |
| TviBrMi | TvvA (1/1) | Tvv2 (1/1) | cattle | Brazil, MS | 1997 | FM876220  - | DQ316047, DQ316048 | 8 |
| TviBrRS1 | TvvB (4/4) | Tvv2 (2/2) | horse | Brazil, RS | 2009 | KX584757 | KC196564-KC196565 | 8 |
| TviBrRS2 | Nd | Tvv2 (1/1) | cattle | Brazil, RS | 2009 | - | KC196543 | 8 |
| TviVzAp1 | Nd | Tvv2 (5/5) | cattle | Venezuela, Apu | 2006 | - | KC196583-KC196587 | 8 |
| TviVzAp2 | Nd | Tvv2 (3/3) | sheep | Venezuela, Apu | 2006 | - | KC196588-KC196590 | 8 |
| TviVzAp3 | Nd | Tvv2 (3/3) | sheep | Venezuela, Apu | 2006 | - | KC196591-KC196593 | 8 |
| TviVzAp4 | Nd | Tvv2 (4/4) | sheep | Venezuela, Apu | 2006 | - | KC196594-KC196597 | 8 |
| TviVzAp5 | Nd | Tvv2 (3/3) | sheep | Venezuela, Apu | 2006 | - | KC196598-KC196600 | 8 |
| TviVzAp6 | Nd | Tvv2 (2/2) | sheep | Venezuela, Apu | 2006 | - | KC196601-KC196602 | 8 |
| TviVzAp7 | Nd | Tvv2 (2/2) | sheep | Venezuela, Apu | 2006 | - | KC196603-KC196604 | 8 |
| TviVzAp9 | Nd | Tvv2 (4/4) | sheep | Venezuela, Apu | 2006 | - | KC196605-KC196608 | 8 |
| TviVzAp10 | TvvA (4/4) | Tvv2 (4/4) | buffalo | Venezuela, Apu | 2015 | KX584750 | - | This study |
| TviVz Ap11 | TvvA (3/3) | Tvv2 (6/6) | buffalo | Venezuela, Apu | 2015 | KX584748 | - | This study |
| TviVzAp12 | TvvA (6/6) | Nd | buffalo | Venezuela, Apu | 2015 | KX584749 | - | This study |
| TviVzAnz1 | Nd | Tvv2 (1/1) | cattle | Venezuela, Anz | 2006 | - | KC196609 | 8 |
| TviVzAnz2 | Nd | Tvv2 (1/1) | cattle | Venezuela, Anz | 2006 | - | KC196610 | 8 |
| TviVzAnz3 | Nd | Tvv2 (2/2) | cattle | Venezuela, Anz | 2006 | - | KC196611-KC196612 | 8 |
| TviVzCoj | Nd | Tvv2 (2/2) | buffalo | Venezuela, Coj | 2006 | - | KC196613-KC196614 | 8 |
| TviVzGu1 | Nd | Tvv2 (2/2) | buffalo | Venezuela, Gua | 2006 | - | KC196615-KC196616 | 8 |
| TviVzGu2 | Nd | Tvv2 (3/3) | buffalo | Venezuela, Gua | 2006 | - | KC196617-KC196619 | 8 |
| AF053744 | TvvA (1/1) | Tvv2 (1/1) | cattle | Venezuela | - | AF053744 | - | 25 |
| TviGuyane | TvvB (1/1) | Tvv2 (4/4) | cattle | French Guiana | 1986 | KX584766 | KC196579-KC196582 | 58 |
| TviColômbia | TvvB (4/4) | nd | cattle | Colômbia | 2014 | KX584754 | - | This study |

Tvv = Lineage *T. vivax.* Brazilian *S*tates: PA, Para; PB, Paraiba; MS, Mato Grosso do Sul; RS, Rio Grande do Sul; Venezuelan Provinces AP, Apure; ANZ, Anzoátegui; COJ, Cojedes; GUA, Guarico. Nd, Not determined.
