# Supplementary figures and images for: New insights from Gorongosa National Park and Niassa National Reserve of Mozambique increasing the genetic diversity of Trypanosoma vivax and Trypanosoma vivax-like in tsetse flies, wild ungulates and livestock from East Africa
Source: Parasit Vectors. 2017 Jul 17;10:337. doi: 10.1186/s13071-017-2241-2 (PMC5513381; doi:10.1186/s13071-017-2241-2)

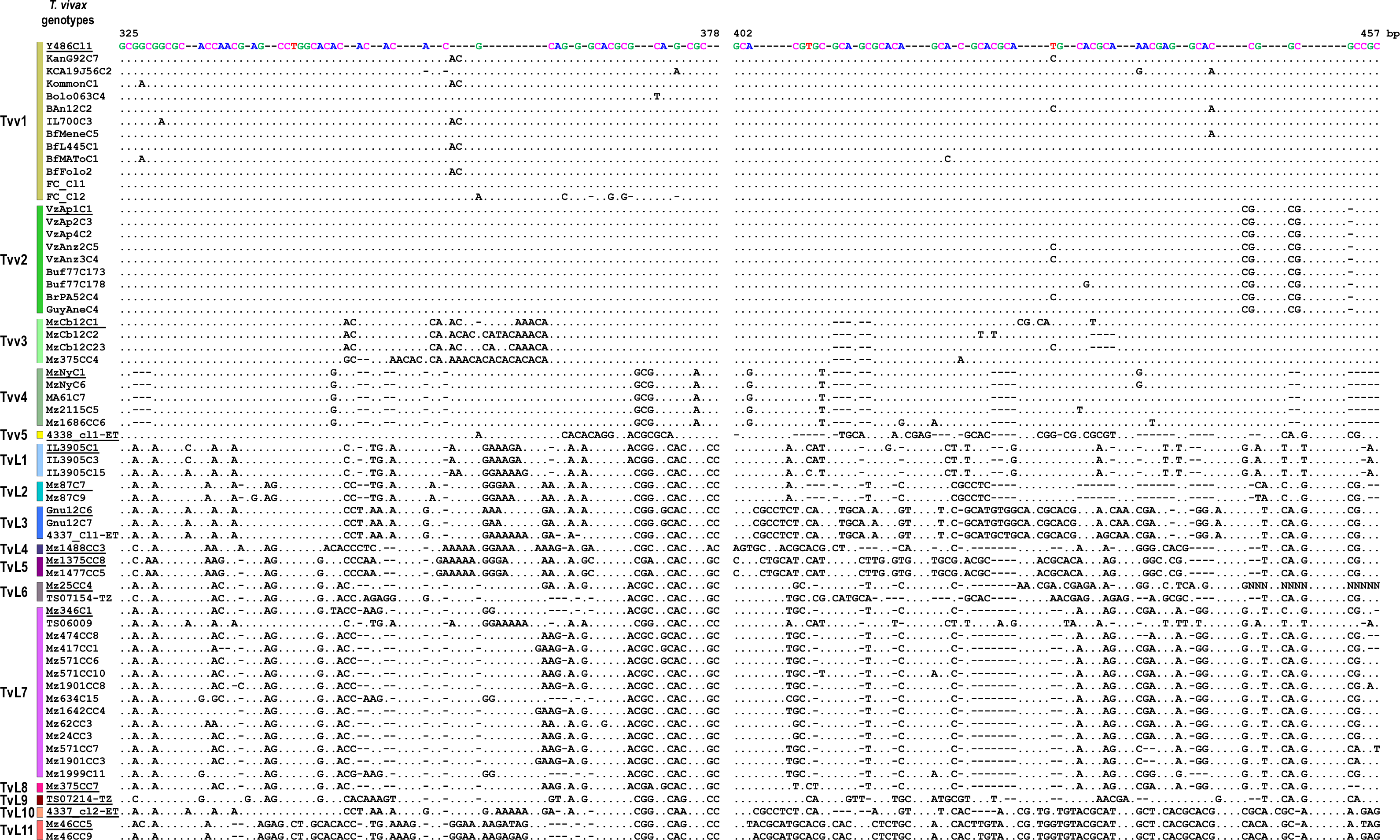

Supplement: Supplementary file 3 — The alignment of ITS1 and ITS2 rDNA sequences from Trypanosoma vivax and T. vivax-like isolates. South American and West African isolates shared highly conserved sequences, exhibiting only punctual polymorphisms. The polymorphic East African isolates revealed blocks of nucleotides that were unique for each genotype, as well as conserved segments shared by closely related genotypes. The reference isolate/sequence representing each genotype is underlined. ITS sequences were deposited in GenBank (accession numbers in Additional files 1 and 2). (TIFF 496 kb) [file 13071_2017_2241_MOESM1_ESM.tif]
